# The Antigen-Presentation Bottleneck in Pediatric Solid Tumors: Developmental Antigens, MHC-I Suppression, and Therapeutic Restoration of Tumor Immune Recognition

**DOI:** 10.3390/biom16101448

**Published:** 2026-10-04

**Authors:** Wasim Iyad Ibrahim Alghoul, Zain Al-Abdeen Mohammed Qassim, Riza Jazlin Neyyappadath, Lakshmi Sudhakaran, Hansiddh Dasgupta, Noor Jawad Kadhim, Imran Rangraze

**Affiliations:** 1 RAK College of Medical Sciences, RAK Medical and Health Sciences University (RAKMHSU), Ras Al Khaimah 11172, United Arab Emirates; wasim.21901111@rakmhsu.ac.ae (W.I.I.A.); riza.21901063@rakmhsu.ac.ae (R.J.N.); noor.alezirej@ehs.gov.ae (N.J.K.); 2 Department of Pediatrics, Zulekha Hospital, Dubai 48577, United Arab Emirates; lsudhakaran@zulekhahospitals.com; 3 Department of Internal Medicine, College of Medical Sciences, RAK Medical and Health Sciences University (RAKMHSU), Ras Al Khaimah 11172, United Arab Emirates

**Keywords:** pediatric solid tumors, antigen presentation, MHC class I, HLA, immunopeptidomics, TCR-T therapy, immune evasion, cancer-testis antigens

## Abstract

Background: Pediatric solid tumors respond poorly to T-cell-directed immunotherapy despite expressing developmentally restricted, cancer-testis, fusion-derived, and lineage-associated antigens, suggesting that therapeutic failure may reflect defective conversion of intracellular antigen expression into surface peptide–major histocompatibility complex class I (pMHC-I) display. This review examines the antigen-presentation bottleneck as a framework for tumor immune invisibility and therapeutic resistance. Methods: We conducted a narrative mechanistic synthesis of pediatric and translational evidence on antigen repertoires, MHC-I and antigen-processing machinery dysfunction, mechanisms of presentation loss, therapeutic restoration, pMHC-directed immunotherapy, and MHC-independent bypass strategies. Results: Antigen expression does not reliably predict natural presentation. Presentation failure may result from HLA loss, coordinated transcriptional repression, developmental and epigenetic states, altered trafficking, SUMOylation, and autophagy-mediated MHC-I degradation. Potentially reversible defects can be targeted through cytokine signaling, NF-κB/IRF1/NLRC5 reactivation, epigenetic or lineage reprogramming, and inhibition of post-translational MHC-I loss; irreversible defects favor MHC-independent surface targeting. Conclusions: Pediatric solid tumors may be antigen-invisible rather than antigen-poor. Therapeutic matching should assess antigen abundance, HLA integrity, antigen-processing competence, natural peptide presentation, quantitative pMHC density, and spatial and longitudinal heterogeneity, then restore presentation or deliberately bypass it. The framework distinguishes the presentation phenotype (quantitative pMHC insufficiency versus qualitative loss or functional absence of a required HLA/APM element) from the underlying mechanism (soft/potentially reversible versus hard/structural) because these dimensions have different biomarker and therapeutic implications.

## 1. Introduction

Pediatric solid tumors remain among the most difficult childhood malignancies to treat with immunotherapy. In contrast to many adult epithelial cancers, they are typically characterized by low tumor mutational burden, recurrent structural and transcriptional drivers, limited spontaneous neoantigen generation, and weak pre-existing antitumor T-cell responses [1,2,3]. These features help explain why immune-checkpoint blockade has shown limited activity in most unselected pediatric solid tumors and why effective T-cell-based approaches require strategies adapted to the distinct biology of childhood cancer [1,2,3].

Low mutational burden, however, does not equate to an absence of therapeutically relevant antigens. Large-scale pediatric transcriptomic analyses have identified tumor-selective surface proteins, lineage-associated transcription factors, and cancer-testis antigens across multiple histologies, with PRAME emerging as a recurrent target in neuroblastoma, osteosarcoma, Ewing sarcoma, and rhabdomyosarcoma [2]. Exon-level profiling has further expanded this repertoire by identifying cancer-specific and alternatively spliced exons with restricted expression in normal tissues, providing additional candidate targets in tumors driven more by developmental dysregulation than by high mutational burden [4].

The strongest evidence for therapeutic relevance comes from direct interrogation of the immunopeptidome. In neuroblastoma, integrated genomic and immunopeptidomic profiling identified recurrent HLA-presented peptides derived from developmentally restricted proteins, including PHOX2B, linking lineage identity to a targetable peptide–MHC landscape [5]. In medulloblastoma, profiling of 28 tumors demonstrated a substantial repertoire of naturally presented HLA class I and II antigens, although most were molecular-subgroup specific [6]. Similarly, Ewing sarcoma studies have identified HLA-A*02:01-restricted LOXHD1 peptides that can be recognized by engineered T cells, illustrating how oncofusion-regulated lineage antigens can generate actionable peptide–MHC targets [7].

These findings expose a critical distinction between antigen expression and antigen presentation. Effective CD8+ T-cell recognition requires not only the expression of a tumor-associated protein, but also its processing, transport, peptide loading, and stable display within an appropriate HLA class I complex. Across advanced pediatric solid tumors, HLA class I expression is heterogeneous, and recurrent transcriptional suppression of HLA-B and TAP genes can restrict antigen presentation despite the persistence of potentially targetable antigens [1]. Direct immunopeptidomic studies further show that RNA or protein abundance correlates imperfectly with the peptides actually displayed at the tumor-cell surface, underscoring the limitations of antigen discovery based on expression alone [6].

Importantly, several presentation defects appear biologically reversible. In neuroblastoma, epigenetic modulation can induce a more immunogenic lineage state, increase MHC-I and antigen-processing machinery, and enhance antigen-specific T-cell recognition [8]. Autophagy inhibition can likewise increase MHC-I expression and improve T-cell-mediated antitumor activity [9], while cytokine stimulation can augment MHC-I and immunogenic surface markers in pediatric sarcoma models [10]. Clinically, PRAME-directed TCR therapy has demonstrated that naturally presented HLA-restricted tumor antigens can be therapeutically exploited when antigen expression and presentation are both sufficient [11].

Together, these data support an antigen-presentation bottleneck in pediatric solid tumors: relevant antigens may be present, yet their therapeutic value is constrained by whether they are processed and displayed as sufficiently abundant peptide–MHC-I complexes for effective T-cell recognition. This review therefore examines the antigenic repertoire of pediatric solid tumors, the molecular mechanisms that suppress MHC-I-dependent tumor visibility, and emerging strategies to restore or bypass defective antigen presentation to enable rational T-cell-directed therapy. Operationally, we treat the bottleneck as an umbrella presentation phenotype and distinguish two separate dimensions: whether failure is quantitative or qualitative at the pMHC level, and whether its underlying mechanism is soft/potentially reversible or hard/structural.

## 2. Literature Search Strategy

This narrative review was informed by a structured literature search designed to capture evidence linking tumor antigen availability, MHC class I (MHC-I) antigen presentation, immune escape, and therapeutic restoration of tumor visibility in pediatric solid malignancies. PubMed/MEDLINE, Scopus, and Web of Science Core Collection were searched from database inception through 20 August 2026. Search terms were combined using Boolean operators and organized around four principal concepts: pediatric solid tumors; tumor-associated and developmentally restricted antigens; MHC-I antigen processing and presentation; and therapeutic manipulation of tumor immune recognition. Representative terms included “pediatric solid tumor”, “childhood cancer”, “neuroblastoma”, “Ewing sarcoma”, “osteosarcoma”, “rhabdomyosarcoma”, “medulloblastoma”, “Wilms tumor”, “tumor antigen”, “cancer-testis antigen”, “PRAME”, “MAGE”, “PHOX2B”, “fusion antigen”, “HLA class I”, “MHC class I”, “B2M”, “TAP1”, “TAP2”, “ERAP”, “NLRC5”, “antigen-processing machinery”, “immunopeptidomics”, “HLA peptidome”, “peptide–MHC”, “immune evasion”, “TCR-T”, “epigenetic regulation”, “autophagy”, and “SUMOylation”. Reference lists of relevant primary studies and major reviews were additionally screened to identify mechanistically important publications not retrieved through the initial searches.

To ensure explicit disease-level coverage and to address entities that were less visible in the mechanism-centered synthesis, targeted PubMed/MEDLINE searches were additionally performed through 20 August 2026 for “hepatoblastoma”, “retinoblastoma”, “nephroblastoma”/“Wilms tumor”, “germ cell tumor”, “germinoma”, “yolk sac tumor”, “atypical teratoid/rhabdoid tumor”, “diffuse midline glioma”, “pediatric high-grade glioma”, and “ependymoma”, combined with terms for HLA/MHC-I, B2M, TAP/APM, immunopeptidomics, and antigen presentation. No major pediatric solid-tumor entity was excluded a priori on the basis of histology. Because this is a mechanism-focused narrative review rather than a systematic histology-by-histology survey, dedicated disease subsections are weighted toward entities for which direct tumor-cell evidence of MHC-I/APM status, natural pMHC display, or presentation restoration is available. Tumor types supported only by small historical series, preclinical or mixed-age data, or indirect evidence are identified explicitly as evidence gaps rather than assumed to share the presentation phenotype of better-studied tumors.

Peer-reviewed original research was prioritized, particularly pediatric tumor studies, immunopeptidomic analyses, mechanistic investigations of MHC-I regulation, and translational or clinical studies evaluating antigen-specific T-cell therapies. Adult cancer studies were included selectively when they provided direct mechanistic evidence for pathways relevant to pediatric tumor biology, including transcriptional, epigenetic, or post-translational regulation of MHC-I. High-quality reviews were used primarily for contextual synthesis and the identification of additional primary literature. Studies lacking direct relevance to tumor antigenicity, antigen-processing or presentation biology, immune recognition, or therapeutic restoration or bypass of the presentation pathway were excluded. Evidence was synthesized narratively according to biological mechanism and translational relevance rather than by formal quantitative pooling.

To prevent cross-age mechanistic evidence from being interpreted as pediatric validation, evidence was interpreted using an explicit hierarchy: pediatric clinical evidence; pediatric patient-tissue or patient-derived evidence; pediatric preclinical models; mixed-age/adolescent-and-young-adult evidence; and, finally, non-pediatric mechanistic evidence. Non-pediatric studies were included to support biological plausibility or nominate conserved mechanisms, but were not used to infer the prevalence of a defect, its effect size, or therapeutic efficacy in children. Accordingly, cross-age evidence was identified as such at the first discussion and framed as hypothesis-generating unless pediatric-specific validation was available. For the therapeutic-index analysis in Section 7.6, targeted safety searches were additionally performed through 20 August 2026 using the intervention classes discussed in this review (HDAC/epigenetic therapy, EZH2/PRC2 inhibition, autophagy inhibition, SUMOylation inhibition, IFN-γ, and TNF) combined with “pediatric”, “phase I”, “safety”, “toxicity”, “adverse event”, “secondary malignancy”, and “normal tissue”. Pediatric clinical data were prioritized when available; adult first-in-human evidence was used only when pediatric safety data were absent. For study-level interpretation, we additionally distinguished the experimental setting of the evidence—human patient-tissue observational, in vitro mechanistic, in vivo preclinical, translational, and clinical—because these categories address different questions and should not be weighted interchangeably. These descriptors were used narratively to clarify evidentiary context and are not intended as a formal quality-grading scale. To address the clinical implementation of immunopeptidomics, targeted methodological searches were also performed for combinations of “immunopeptidomics” with “low-input”, “small biopsy”, “tumor cellularity”, “data-independent acquisition”, “DIA”, “microfluidic”, “PASEF”, “high-throughput”, “validation”, and “standardization”.

## 3. The Antigenic Repertoire of Pediatric Solid Tumors

### 3.1. Developmental, Lineage-Restricted, and Oncofetal Antigens

The antigenic repertoire of pediatric solid tumors is shaped less by extensive mutational diversification than by persistence or reactivation of developmental programs. This creates a class of nonmutated targets whose expression is linked to cell lineage or fetal development but is limited in most postnatal tissues. Neuroblastoma provides a compelling example: integrated genomic and immunopeptidomic profiling identified recurrent HLA-presented peptides derived from proteins involved in noradrenergic differentiation and sympathetic nervous-system development, with PHOX2B prioritized as a highly tumor-restricted developmental target [5].

This principle extends across pediatric malignancies. Exon-level profiling identified oncofetal candidates including alternatively spliced FN1 and TNC isoforms and gene-level targets such as TGFB2, WNT5A, GPC3, and IGF2, many of which show restricted expression beyond fetal development [4]. GPC2 illustrates how the same developmental biology can generate a therapeutically accessible surface antigen: it is highly expressed in most neuroblastomas, has limited expression in normal childhood tissues, and contributes to tumor-cell maintenance [12]. However, subsequent CAR-T studies showed that efficacy is strongly conditioned by antigen density, emphasizing that developmental specificity alone does not ensure therapeutic accessibility [13].

### 3.2. Cancer-Testis Antigens

Cancer-testis antigens provide a second major source of nonmutated tumor-associated targets. Among these, PRAME is particularly prominent. In neuroblastoma, PRAME expression was highly prevalent and was observed in all advanced-stage tumors in one primary cohort, supporting its relevance as a broadly shared antigen [14]. Pan-pediatric profiling subsequently confirmed PRAME expression across several extracranial pediatric solid-tumor types [2]. In osteosarcoma, PRAME was detected in more than 70% of patient specimens, while high expression was associated with poorer prognosis and lung metastasis [15].

The MAGE family illustrates the same concept in rhabdomyosarcoma. MAGE-A-positive rhabdomyosarcoma cells were shown not only to express these antigens, but also to process them into peptides recognizable by HLA-restricted T cells when sufficient HLA expression was present [16]. This distinction between antigen abundance and antigen presentation is important because cancer-testis antigen expression alone does not establish that a tumor is functionally visible to T cells.

### 3.3. Fusion-Associated, Oncofusion-Regulated, and Splice-Derived Antigens

Fusion-driven pediatric tumors offer an additional route to tumor selectivity. Ewing sarcoma is characterized by recurrent EWSR1–ETS fusions, most commonly EWSR1–FLI1. Peptides spanning the EWSR1–FLI1 fusion region have demonstrated immunogenic potential; although native peptides showed limited HLA-A2.1 stabilization, anchor-modified peptides generated cytotoxic T cells capable of recognizing tumor cells presenting the endogenous antigen [17].

Tumor selectivity can also arise indirectly from transcriptional programs imposed by oncogenic fusions. LOXHD1 is regulated by EWSR1::FLI1 and exhibits restricted normal-tissue expression. Immunopeptidomic analysis identified three HLA-A*02:01-restricted LOXHD1 epitopes, one of which enabled the isolation of a TCR capable of mediating cytotoxicity against Ewing sarcoma cells [7]. Alternative splicing further broadens this repertoire: among the 157 candidate pediatric targets identified by exon-level analysis, nine arose from alternative splicing, including the FN1, TNC, and COL6A3 isoforms validated in osteosarcoma [4].

### 3.4. Antigen Diversity Does Not Equal Therapeutic Visibility

Collectively, these studies demonstrate that pediatric solid tumors are not uniformly antigen-poor; rather, they contain a biologically diverse but heterogeneous repertoire of developmental, cancer-testis, fusion-associated, and splice-derived targets. The critical distinction is how those antigens are accessed therapeutically. Surface antigens such as GPC2 can be recognized directly by CARs or antibodies and therefore bypass conventional HLA-dependent processing [12,13]. In contrast, intracellular targets such as PHOX2B, PRAME, MAGE, and LOXHD1 are therapeutically available to conventional TCR-based immunity only when they are efficiently processed and displayed as peptide–HLA complexes [5,7,16]. These distinct routes of antigen accessibility and therapeutic recognition are summarized in Figure 1. This distinction shifts the focus from antigen expression alone to the more consequential question of antigen presentation, which forms the central bottleneck addressed in the following section.

## 4. From Antigen Expression to Immune Visibility: The Antigen-Presentation Bottleneck

### 4.1. The MHC-I Antigen-Processing Pathway

For intracellular tumor antigens, expression is only the first step toward immune recognition. Cytosolic proteins must be degraded by the proteasome or immunoproteasome, after which resulting peptides are transported into the endoplasmic reticulum by TAP1/TAP2, loaded onto a complex formed by an MHC-I heavy chain and β2-microglobulin, and transported to the cell surface for surveillance by cytotoxic T lymphocytes [18]. Disruption at any point in this pathway can therefore uncouple intracellular antigen abundance from functional T-cell recognition.

This distinction is particularly relevant to pediatric solid tumors, in which candidate developmental and cancer-testis antigens can coexist with deficient antigen-processing machinery. In a large cohort of advanced pediatric cancers, Lim et al. demonstrated heterogeneous HLA-I expression accompanied by recurrent suppression of HLA-B, TAP1, and TAP2, with low B2M expression also observed in several tumor types [1]. Thus, the biologically relevant unit for T-cell therapy is not the tumor-associated protein itself, but the peptide–HLA complex ultimately displayed at the tumor-cell surface.

### 4.2. Expression Does Not Predict Presentation

Transcriptomic and proteomic approaches remain valuable for identifying tumor-selective proteins, but neither reliably establishes that a candidate antigen enters the HLA ligandome. Direct evidence from medulloblastoma demonstrates a poor correlation between precursor RNA or protein abundance and corresponding HLA-bound peptides, limiting the prediction of actionable epitopes from expression data alone [6]. Immunopeptidomic profiling of 28 primary medulloblastomas consequently revealed thousands of naturally presented HLA-I peptides, but only a restricted subset satisfied tumor-association criteria, and most candidate antigens were not uniformly shared across molecular subgroups [6].

Neuroblastoma provides an especially clear demonstration of this disconnect. Earlier tumor analyses identified the expression of MAGE-A1, MAGE-A3/A6, and NY-ESO-1, yet the examined antigen-positive specimens lacked detectable MHC-I expression, exposing a situation in which intracellular antigen availability did not translate into T-cell accessibility [19]. More directly, PRAME-expressing neuroblastoma cells with low endogenous HLA-I were poorly recognized by PRAME-specific cytotoxic T cells, whereas increasing HLA-A2 expression restored presentation of the PRAME-derived SLLQHLIGL peptide and enabled antigen-specific recognition [20].

### 4.3. Operational Definition and Two-Axis Taxonomy of the Antigen-Presentation Bottleneck

In this review, the antigen-presentation bottleneck is used in a restricted operational sense to denote any tumor-cell constraint between intracellular availability of an HLA-restricted antigen and productive recognition of the corresponding surface peptide–MHC-I (pMHC-I) complex. It therefore does not denote antigen scarcity itself, nor is it synonymous with any single HLA or antigen-processing machinery (APM) abnormality; rather, it describes failure to convert an available intracellular antigen into therapeutically accessible pMHC-I display.

Two independent axes are required to classify this failure. On the phenotypic axis, a quantitative deficit denotes preservation of the relevant pMHC species but at a density below the threshold required for productive receptor engagement, whereas a qualitative deficit denotes loss or functional absence of a required element of the recognition chain—such as tumor-specific loss of the restricting HLA allele or loss/inactivation of an indispensable APM component—such that the relevant pMHC species cannot be generated or displayed. For the purposes of this review, constitutional absence of the HLA allele required by a given TCR therapy is classified as an HLA-eligibility mismatch rather than a tumor-acquired presentation defect [1,11,21].

On the mechanistic axis, either quantitative or qualitative presentation failure may reflect a soft defect, in which the relevant pathway remains structurally intact but is transcriptionally, epigenetically, trafficking-, or degradation-mediated suppressed, or a hard defect, in which structural genomic loss or other fixed disruption makes restoration unlikely through pathway induction alone. These axes should not be conflated: quantitative versus qualitative describes what is missing at the pMHC level, whereas soft versus hard describes why it is missing and whether restoration is biologically plausible. This two-axis framework is used throughout the subsequent sections to link mechanism, biomarker interpretation, and therapy selection [1,8,9,22,23,24].

### 4.4. Immunopeptidomics Defines Functional Tumor Visibility

Mass-spectrometry-based immunopeptidomics therefore provides a critical bridge between antigen discovery and therapeutic validation because it measures peptides that have successfully traversed the processing pathway and reached HLA molecules. This principle has enabled the direct identification of actionable pMHC targets in pediatric tumors including the PHOX2B-derived QYNPIRTTF peptide in neuroblastoma [5] and HLA-A*02:01-restricted LOXHD1 epitopes in Ewing sarcoma [7].

Yet presentation is not simply binary. The abundance of a particular pMHC complex and the functional avidity of the recognizing T-cell receptor jointly influence whether presentation becomes productive cytotoxicity. PRAME-specific T-cell clones required markedly different peptide concentrations for equivalent activation, demonstrating that receptor avidity strongly influences the pMHC density required for productive recognition [21].

Within this framework, pMHC abundance remains a quantitative continuum when the required HLA and APM elements are retained, but not all antigen-presentation bottlenecks are quantitative. A tumor may instead have a qualitative presentation failure in which the relevant pMHC species cannot be generated because a necessary HLA/APM element is absent or functionally unavailable. Pediatric tumors may therefore possess therapeutically attractive antigens yet remain functionally invisible either because pMHC density is insufficient or because presentation competence is qualitatively disrupted. Establishing which phenotype is present requires the direct assessment of HLA allelic integrity, antigen-processing competence, natural pMHC display, and where feasible, pMHC abundance.

The translational value of direct pMHC measurement must nevertheless be interpreted in light of analytical sensitivity. In particular, non-detection in a small pediatric biopsy cannot automatically be equated with biological absence of the relevant pMHC species. The low-input limitations of immunopeptidomics and emerging strategies for standardized clinical implementation are considered in Section 10.2.

## 5. MHC-I and Antigen-Processing Dysfunction Across Pediatric Solid Tumors

### 5.1. Pan-Pediatric Patterns of Presentation Failure

Available pediatric data indicate that defective antigen presentation is not a uniform loss-of-function phenotype, but a spectrum ranging from locus-specific HLA alterations to the coordinated suppression of antigen-processing machinery. In advanced pediatric solid tumors, somatic mutations affecting HLA or antigen-processing genes were uncommon outside genetically unstable or mismatch-repair-deficient tumors, whereas copy-number changes, HLA loss of heterozygosity (LOH), and transcriptional suppression were more prominent [1]. HLA-I LOH occurred in approximately 5.9–7.7% of tumors overall but was enriched in osteosarcoma and glioblastoma, while HLA-I loss was frequently associated with reduced HLA-B and TAP expression [1].

This distinction is therapeutically important: complete destruction of the presentation pathway and reversible suppression are unlikely to respond to the same intervention. Pan-pediatric profiling further showed HLA-B, TAP1, and TAP2 transcript levels below those of control tissues in approximately 80% of examined patient-derived xenografts, with low B2M also observed in neuroblastoma, nephroblastoma, and most rhabdomyosarcoma models [1]. Notably, low HLA-B transcription could coexist with apparently preserved pan-HLA-I immunohistochemical staining, suggesting that global HLA staining may underestimate functionally relevant locus-specific defects.

### 5.2. Neuroblastoma: Coordinated Suppression of the Presentation Machinery

Neuroblastoma represents the clearest pediatric model of coordinated MHC-I/APM suppression. Aggressive neuroblastoma cell lines exhibit low MHC-I together with reduced expression of antigen-processing components, including endoplasmic-reticulum aminopeptidases, resulting in inefficient tumor-antigen presentation to cytotoxic T cells [22]. Low IRF1 and NF-κB p65 expression is associated with this phenotype, indicating that several components of the pathway can be simultaneously constrained by upstream regulatory programs rather than by independent structural defects [22].

### 5.3. Ewing Sarcoma and Rhabdomyosarcoma: Heterogeneous but Potentially Reversible Defects

Ewing sarcoma also demonstrates substantial impairment of HLA-I expression. Berghuis et al. found complete or partial HLA-I absence in 79% of 67 tumors, with lung metastases consistently lacking detectable HLA-I and sequential specimens showing a tendency toward reduced expression during progression [25]. Importantly, IFN-γ induced TAP1/2, tapasin, immunoproteasome components, β2-microglobulin, and HLA-I in Ewing sarcoma cell lines, supporting a major contribution from regulatory suppression rather than irreversible pathway loss [25].

Rhabdomyosarcoma is less comprehensively characterized, but pan-pediatric profiling identified low B2M and frequent suppression of HLA-B and TAP transcripts in examined models [1]. The comparatively limited mechanistic dataset in rhabdomyosarcoma underscores an important evidence gap rather than establishing a uniform presentation phenotype.

### 5.4. Osteosarcoma: Context-Dependent and Spatially Heterogeneous HLA-I Expression

Osteosarcoma illustrates particularly well why HLA-I status should not be reduced to a binary tumor-type classification. Tsukahara et al. reported HLA-I loss or downregulation in 52% of primary osteosarcomas and 88% of metastatic lesions, with high HLA-I expression associated with better overall and event-free survival [26].

Conversely, Sundara et al. observed frequent HLA-I expression in both primary and metastatic osteosarcoma and found that tumor-infiltrating T cells spatially colocalized with HLA-I-positive tumor regions [27]. These apparently discordant findings likely reflect differences in cohort composition, disease context, assay methodology, and intratumoral immune pressure, and collectively argue for patient-level and spatial assessment rather than histology-wide assumptions.

Broader sarcoma data reinforce this principle. A 2024 study of 126 bone and soft-tissue sarcomas demonstrated defects in at least one antigen-processing component across all examined histologies, with substantial variation between histotypes, individual patients, and tumor regions [28]. Although not pediatric-specific, these findings support the concept that presentation failure may involve distinct combinations of HLA and APM abnormalities rather than uniform loss of a single component.

Importantly, spatial heterogeneity provides an additional explanation for the apparently discordant osteosarcoma literature. Sundara et al. showed that tumor-infiltrating T cells colocalized with HLA-I-positive tumor regions [27], while broader sarcoma profiling demonstrated region-to-region variation in HLA and antigen-processing components [28]. Together, these observations raise the possibility that HLA-I-positive and HLA-I-low/negative compartments may coexist within the same tumor. Under this model, the difference between the findings of Tsukahara et al. and Sundara et al. need not reflect only cohort composition or assay methodology: a single block, core biopsy, or sampled metastatic focus could classify the tumor according to the local compartment captured and therefore incompletely represent whole-tumor presentation competence. This remains a biologically plausible interpretation rather than a demonstrated explanation for the discrepancy between those two osteosarcoma cohorts, because exhaustive multiregion mapping within the same lesions was not performed [26,27,28].

This sampling problem has direct implications for biomarker-driven patient selection. When regional heterogeneity is suspected, a single pan-HLA-I result should not be assumed to represent all tumor compartments. Where tissue availability permits, assessment should incorporate multiple regions and/or paired primary and metastatic sites, together with allele-specific HLA/APM evaluation and functional pMHC measurement when feasible. Otherwise, a patient could be classified as presentation-competent or presentation-deficient on the basis of the particular region sampled, even when therapeutically relevant discordant compartments coexist [27,28].

### 5.5. Medulloblastoma: Developmental State Intersects with Immune Presentation

Medulloblastoma represents a distinct presentation context in which HLA-I expression may reflect developmental lineage state as well as immune selection. Smith et al. found heterogeneous HLA-I expression and proposed that this pattern could partly mirror the developmental HLA program of cerebellar precursor cells rather than exclusively represent immune-edited HLA-loss variants [29]. HLA-I molecules in medulloblastoma may also participate in signaling functions independent of antigen presentation, including ERK1/2 activation, further complicating the interpretation of HLA expression solely as an immune-evasion marker [29].

### 5.6. Less-Characterized Pediatric Solid Tumors: Available Evidence and Explicit Gaps

Wilms tumor/nephroblastoma has more presentation-related evidence than was reflected in the original version of this review. In an immunohistologic study of six Wilms tumors, blastemal cells were class I MHC antigen-negative whereas more differentiated tumor structures were positive, suggesting that MHC-I expression can vary with developmental differentiation state [30]. Consistent with this, pan-pediatric profiling detected low B2M expression in nephroblastoma models [1]. These observations complement the later clinical demonstration that PRAME-specific TCR-T can be active in an HLA-A*02:01-positive nephroblastoma [31], but the available evidence remains too small to establish a uniform HLA/APM phenotype across Wilms tumors.

Retinoblastoma also has direct tissue-level evidence of presentation-pathway abnormalities. In 30 archival tumors, HLA class I, B2M, and multiple class I antigen-processing components, including LMP2/LMP10, TAP1, tapasin, and calnexin, were reduced in tumors with choroidal or optic-nerve invasion compared with non-invasive tumors [32]. These findings support coordinated impairment of the presentation pathway in aggressive retinoblastoma, although contemporary immunopeptidomic studies and mechanism-matched restoration experiments remain limited.

For hepatoblastoma, presentation-focused evidence is more limited and is predominantly preclinical. Human hepatoblastoma cell lines showed low HLA class I expression in an analysis of NK-cell recognition and activating ligands [33]. This observation is compatible with reduced adaptive immune visibility, but it does not establish a hepatoblastoma-wide presentation phenotype in patient tumors; hepatoblastoma should therefore be treated as an incompletely characterized entity rather than assigned the neuroblastoma-like MHC-I-low phenotype by analogy.

Among the CNS tumors beyond medulloblastoma, atypical teratoid/rhabdoid tumor (AT/RT) provides direct evidence that naturally presented HLA ligands can be recovered despite the low-mutational-burden biology of this disease. Mass-spectrometry analysis of 23 AT/RTs identified naturally presented HLA class I and II ligands, including tumor-exclusive and non-canonically derived peptides [34]. Diffuse midline glioma (DMG) has also entered the immunopeptidomic literature: a 2026 study using human DMG xenograft models confirmed surface HLA-I expression and a measurable MHC-I ligandome whose composition changed after EGFR/PI3K inhibition [35]. These findings argue against treating pediatric CNS tumors as uniformly presentation-deficient; however, the evidence is entity- and model-specific, and ependymoma and several other CNS histologies remain incompletely characterized with respect to tumor-cell HLA/APM function.

Pediatric germ-cell tumors remain particularly heterogeneous and underdefined in this framework. Recent analysis of malignant ovarian germ-cell tumors reported loss of MHC class I expression across the dysgerminoma, yolk-sac tumor, and immature-teratoma subtypes [36]; however, that cohort was not designed as a pediatric-only series. We therefore treat these findings as supportive evidence for a presentation defect in germ-cell tumor biology, not as proof of a uniform phenotype in childhood germ-cell tumors. Accordingly, Section 5.1, Section 5.2, Section 5.3, Section 5.4 and Section 5.5 should be read as evidence-rich exemplars rather than a complete catalogue of pediatric solid-tumor histologies; absence of a dedicated mechanistic subsection for a given entity reflects insufficient direct evidence, not exclusion from the conceptual scope of the review.

Taken together, pediatric solid tumors exhibit quantitatively and mechanistically distinct defects in antigen presentation. The clinically relevant question is therefore not simply whether HLA-I is present or absent, but which component of the presentation pathway is impaired, whether the defect is spatially or temporally heterogeneous, and—critically—whether it remains reversible. These distinctions provide the rationale for examining the molecular mechanisms that generate tumor invisibility.

## 6. Molecular Mechanisms of MHC-I Suppression and Tumor Invisibility

MHC-I dysfunction in pediatric solid tumors can arise at several levels, from irreversible genomic loss to reversible transcriptional, epigenetic, and post-translational suppression. Mechanistically separating these abnormalities is critical because an antigen-rich tumor with genetically intact but repressed presentation machinery represents a fundamentally different therapeutic problem from a tumor that has permanently lost the relevant HLA allele or an indispensable antigen-processing component. Accordingly, the hard/soft distinction used below refers to mechanism and reversibility, whereas the quantitative/qualitative distinction introduced in Section 4.3 refers to the resulting presentation phenotype; the two dimensions are related but not interchangeable.

### 6.1. Genetic Disruption: The “Hard” Presentation Defect

Genetic alterations constitute the most structurally constrained form of antigen-presentation failure. Pan-pediatric genomic profiling found somatic mutations affecting HLA and antigen-processing and presentation genes to be uncommon in most tumors, with greater enrichment in mismatch-repair-deficient or genomically unstable cancers [1]. In contrast, HLA loss of heterozygosity (LOH) and copy-number alterations occurred recurrently, with HLA-I LOH particularly enriched in osteosarcoma and glioblastoma [1]. Such alterations can remove specific peptide-binding alleles and thereby eliminate the presentation of otherwise suitable HLA-restricted antigens.

NLRC5 illustrates how alterations in an upstream regulator may generate broader pathway dysfunction. NLRC5 acts as a transcriptional coordinator of HLA-I together with B2M, TAP1, and immunoproteasome components; across cancer datasets, promoter methylation, copy-number loss, and somatic mutations in NLRC5 were associated with coordinated reduction in these genes [37]. Although these observations derive predominantly from adult tumors, they provide a mechanistic framework for interpreting coordinated APM failure when individual HLA genes remain structurally intact. Accordingly, the prevalence, causal importance, and reversibility of NLRC5 dysregulation require direct validation in pediatric tumor tissues and pediatric-specific experimental models before equivalent biological or therapeutic relevance can be assumed in childhood cancers.

### 6.2. Transcriptional Suppression: Coordinated Loss Without Genomic Destruction

The strongest pediatric evidence for transcriptional repression comes from neuroblastoma. Forloni et al. demonstrated that NF-κB, rather than MYCN itself, directly controls MHC-I expression in human neuroblastoma. NF-κB p65 bound MHC-I regulatory regions, while the manipulation of NF-κB altered both MHC-I and the peptide-trimming aminopeptidases ERAP1 and ERAP2, demonstrating coordinated control of antigen processing and presentation [23].

IRF1 cooperates with this pathway. Aggressive neuroblastoma models with low MHC-I also displayed reduced IRF1, NF-κB p65, and multiple APM components; combined restoration of IRF1 and p65 substantially reversed the MHC-I/APM-low phenotype and restored susceptibility to antigen-specific cytotoxic T cells [22]. Upstream CRISPR screening further identified N4BP1 and TNIP1 as suppressors of NF-κB-driven MHC-I expression, with their depletion increasing surface MHC-I across several neuroblastoma cell lines [38]. These data establish transcriptional repression as a prototypical soft defect in which the machinery is retained but insufficiently activated.

### 6.3. Epigenetic and Developmental States Couple Lineage Identity to Immune Visibility

Epigenetic regulation provides a particularly compelling link between developmental origin and MHC-I suppression. In neuroblastoma, HDAC inhibition with entinostat increased the expression of genes involved in antigen processing and presentation and induced an adrenergic-to-mesenchymal lineage transition associated with greater T-cell and NK-cell immunogenicity [8]. The treatment also increased the MHC-I and immunoproteasome components, demonstrating that lineage plasticity and antigen-presentation competence can be mechanistically coupled in a pediatric embryonal tumor [8].

This pediatric observation aligns with a broader developmental mechanism described by Burr et al., in which PRC2 maintains bivalent H3K4me3/H3K27me3 chromatin at MHC-I/APM loci, suppressing basal expression and limiting cytokine responsiveness [39]. Importantly, similar chromatin architecture occurs in embryonic stem cells and neural progenitors, suggesting that cancers derived from primitive developmental states can co-opt a physiological program of low MHC-I expression [39]. WHSC1 provides an additional chromatin-level mechanism: NLRC5-dependent recruitment of WHSC1 promotes H3K36me2 and expression of B2M, MHC-I, and TAP1, whereas WHSC1 loss reduces this program [40]. Importantly, the PRC2 cancer-model evidence in Burr et al. was generated in non-pediatric experimental systems; it is therefore used here as cross-age mechanistic support rather than direct evidence that PRC2-mediated MHC-I/APM silencing is prevalent in pediatric tumors [39]. The developmental parallel with embryonic stem cells and neural progenitors strengthens biological plausibility but does not by itself establish the same tumor-cell state across childhood malignancies.

### 6.4. Post-Translational Removal: Trafficking, SUMOylation, and Autophagy

MHC-I can also be suppressed after transcription. In Ewing sarcoma, APLP2 interacts with MHC-I and promotes reduced surface availability; APLP2 silencing increased cell-surface MHC-I, implicating altered trafficking and internalization in immune evasion [41]. SUMOylation represents another regulatory layer. Activated SUMO signaling stabilizes the transcriptional repressor SAFB and suppresses HLA-A, HLA-B, B2M, TAP1, LMP2, and LMP7, whereas SUMO inhibition reverses this phenotype [18]. The SUMOylation evidence requires the same qualification: Demel et al. established this pathway principally in lymphoma and other cross-cancer model systems, with additional cell-line experiments that included U-2-OS and Kelly cells, but did not establish its prevalence in pediatric patient tumors [18]. We therefore treat SUMO-dependent APM suppression as a mechanistically plausible, cross-tumor pathway requiring pediatric validation rather than as an established pan-pediatric defect.

Autophagy is particularly relevant because pediatric-specific evidence now directly links it to MHC-I loss. In neuroblastoma, both genetic and pharmacological autophagy inhibition increased surface MHC-I, enhanced CD8+ T-cell recruitment, and improved susceptibility to antigen-specific T-cell and GD2-CAR-T-mediated killing [9]. In osteosarcoma, UBA2 was recently identified as a driver of an immune-cold state by promoting SESN2 SUMOylation and autophagic flux; this reduced surface MHC-I through NBR1-mediated autophagy–lysosomal degradation and limited CD8+ T-cell infiltration [24]. This mechanism closely parallels NBR1-dependent autophagic MHC-I degradation previously demonstrated in pancreatic cancer [42], providing mechanistic convergence across tumor types. The pancreatic-cancer study cited for NBR1-dependent MHC-I degradation was an adult PDAC model and is used only to demonstrate mechanistic convergence; it does not establish that this pathway has the same frequency, regulation, or therapeutic relevance in pediatric tumors [42].

Collectively, these mechanisms support a clinically useful distinction between hard defects, dominated by irreversible structural loss such as HLA LOH, and soft defects, in which intact presentation machinery is transcriptionally, epigenetically, or post-translationally suppressed. The latter are especially important because tumor invisibility may represent a reversible cellular state, creating a direct rationale for the therapeutic restoration of antigen presentation.

## 7. Therapeutic Restoration of Antigen Presentation

For tumors with reversible antigen-presentation defects, the therapeutic objective is not merely to increase the total HLA-I expression, but to restore functional peptide–MHC-I display sufficiently to permit antigen-specific T-cell recognition. The mechanism responsible for invisibility should therefore determine the restoration strategy: inflammatory signaling can reactivate inducible pathways, transcriptional or epigenetic interventions can reverse coordinated repression, and inhibition of intracellular degradation can preserve MHC-I at the tumor-cell surface. However, mechanistic reversibility is not sufficient to justify treatment: a restoration strategy must also demonstrate a therapeutic window in which tumor pMHC is increased without unacceptable systemic toxicity or clinically relevant augmentation of the same therapeutic target on normal tissues.

### 7.1. Cytokine-Mediated Restoration: Proof of Reversibility

IFN-γ provides the strongest pediatric evidence that an MHC-I-low phenotype can remain therapeutically reversible. In neuroblastoma, IFN-γ increased MHC-I, rendered previously resistant tumor cells susceptible to patient-derived survivin-specific cytotoxic T cells, and increased T-cell infiltration into tumors [43]. Importantly, a pilot clinical study of five patients with high-risk neuroblastoma showed increased tumor-cell MHC-I expression accompanied by enhanced CD3+ T-cell infiltration in two patients after systemic IFN-γ, whereas nonresponding tumors showed little increase in T-cell infiltration [43]. This heterogeneity provides an early clinical indication that cytokine restoration depends on the preservation of an inducible downstream presentation pathway.

A similar process can arise from local immune pressure. Activated NK cells induced MHC-I on neuroblastoma predominantly through IFN-γ and enabled the presentation of PRAME-derived peptide–MHC-I complexes that were subsequently recognized by PRAME-specific cytotoxic T cells [20]. This is particularly informative because it demonstrates restoration at the level of functional antigen-specific recognition, rather than HLA expression alone.

TNF represents a complementary inflammatory strategy in pediatric sarcomas. Among the several cytokines tested across the osteosarcoma, Ewing sarcoma, and rhabdomyosarcoma models, TNF was the most consistent inducer of MHC-I and ICAM-1 [10]. In Ewing sarcoma, TNF enhanced recognition of responsive HLA-A*02:01-positive cells by antigen-specific CD8+ T cells, with blocking experiments implicating both MHC-I and ICAM-1 [10]. Complementary cytokine-conditioning experiments in Ewing sarcoma similarly increased MHC-I expression and enhanced antigen-specific T-cell activation and cytotoxicity [44]. Responses nevertheless differed markedly among cell lines, arguing against universal cytokine sensitivity and supporting pretherapeutic assessment of inducibility.

### 7.2. Re-Engaging Transcriptional Control of MHC-I

Where presentation failure reflects deficient transcriptional signaling, reactivation of upstream regulators may restore multiple APM components simultaneously. In aggressive neuroblastoma, combined expression of IRF1 and NF-κB p65 substantially reconstituted the antigen-processing pathway and restored MAGE-A3-specific cytotoxic T-cell killing [22]. This supports targeting regulatory nodes rather than individual HLA molecules when suppression involves a coordinated transcriptional program.

NLRC5 represents another potentially important node because it coordinately regulates HLA-I, B2M, TAP1, and immunoproteasome genes [37]. Genetic and epigenetic disruption of NLRC5 is associated with broad MHC-I/APM suppression, making restoration of NLRC5 activity mechanistically attractive [37]. However, direct pharmacologic activation of NLRC5 has not yet been established as a pediatric therapeutic strategy; it should therefore be considered a candidate restoration target rather than a clinically validated intervention. Any therapeutic strategy aimed at reactivating NLRC5 should therefore be regarded as hypothesis-generating in pediatrics until pediatric-specific models demonstrate the restoration of functional pMHC display and an acceptable pediatric safety window.

### 7.3. Epigenetic and Lineage Reprogramming

Epigenetic therapy may be particularly suited to developmental tumors in which immune invisibility is coupled to lineage state. In neuroblastoma, the HDAC inhibitor entinostat increased HLA-I, TAP1/2, and immunoproteasome components across cell lines and patient-derived organoids and enhanced the MHC-I-dependent activation and cytotoxicity of PRAME-specific T cells [8]. These changes occurred alongside an adrenergic-to-mesenchymal lineage transition, indicating that presentation competence can be remodeled together with tumor-cell identity [8].

Broader mechanistic work similarly demonstrates that pharmacologic disruption of PRC2 can reverse coordinated chromatin-mediated silencing of MHC-I/APM genes and re-establish T-cell-mediated antitumor immunity [39]. Although these experiments are not pediatric clinical evidence, they provide a mechanistic rationale for targeting developmentally inherited chromatin programs in MHC-I-low childhood tumors. Given the non-pediatric origin of this PRC2 dataset, we regard PRC2 inhibition as a candidate restoration principle that requires pediatric tumor-specific validation, not as evidence that PRC2 blockade will restore functional pMHC display in children.

### 7.4. Preventing Post-Translational Loss of MHC-I

An alternative strategy is to preserve MHC-I after synthesis. In neuroblastoma, genetic and pharmacologic autophagy inhibition increased surface MHC-I and enhanced PRAME-specific T-cell cytotoxicity [9]. In osteosarcoma, UBA2 promoted SESN2 SUMOylation and autophagic flux, leading to NBR1-dependent lysosomal degradation of MHC-I; pharmacologic UBA2 inhibition with ML-792 reduced tumor growth and sensitized tumors to immune-checkpoint blockade [24]. SUMO-pathway inhibition likewise increased the MHC-I/APM components and peptide–MHC display in experimental cancer models, providing another potentially reversible post-transcriptional axis [18]. However, although SUMO-pathway inhibition provides a coherent mechanistic strategy, its ability to restore therapeutically relevant pMHC display in pediatric tumors has not yet been established and should be considered investigational in this context [18].

### 7.5. Restoration Should Be Mechanism-Matched and Functionally Verified

Restoration strategies should therefore be selected according to the underlying lesion rather than applied as nonspecific MHC-I enhancers. Moreover, inflammatory activation is not uniformly beneficial: prolonged interferon signaling can generate STAT1-associated epigenomic remodeling and multiple inhibitory programs that promote adaptive resistance, although this evidence derives predominantly from adult cancer models [45]. Benci et al. established the chronic interferon-resistance program primarily in melanoma-centered murine models, with correlative data from adult melanoma patients, rather than in pediatric cancers [45]. We therefore do not assume that prolonged IFN signaling produces an identical adaptive-resistance state across childhood tumors. Pediatric data already demonstrate marked heterogeneity in cytokine inducibility across neuroblastoma and sarcoma models [10,43,44]; consequently, the balance between acute restoration of antigen presentation and chronic adaptive resistance should be defined empirically for each pediatric histology, cytokine, dose, and exposure schedule.

Most importantly, therapeutic restoration should not be defined by increased HLA-I staining alone. The relevant endpoint is the recovery of naturally processed peptide–MHC complexes at a density sufficient for antigen-specific recognition. Restoration should therefore be temporally integrated with TCR- or pMHC-directed therapy, whereas tumors with irreversible presentation defects may require deliberate MHC-independent bypass strategies. Mechanism matching should therefore first establish whether presentation failure is quantitative or qualitative and then determine whether the causative lesion is soft or hard. Quantitative or qualitative defects caused by regulatory suppression may remain candidates for restoration, whereas structural loss of the restricting HLA allele or another indispensable presentation element is unlikely to be corrected by nonspecific HLA induction and instead favors an MHC-independent strategy. Spatial heterogeneity must also qualify mechanism matching: evidence that a presentation defect is reversible in one sampled region does not establish that all regions or metastatic deposits share the same defect. When HLA/APM abnormalities vary across tumor regions, restoration strategies should therefore be interpreted against spatially resolved biomarker data rather than a single-biopsy classification [28].

### 7.6. Therapeutic Index and Pediatric-Specific Safety Considerations

Presentation restoration should be evaluated as a pharmacologic priming strategy, not simply as a method for maximizing HLA-I. HDAC/PRC2 inhibitors, autophagy inhibitors, SUMOylation modulators, and inflammatory cytokines act on pathways that are also active in normal cells. Consequently, systemic exposure can produce conventional organ toxicities and may also alter antigen processing or HLA expression outside the tumor. For HLA-restricted therapies, the safety-relevant endpoint is therefore the change in the specific therapeutic pMHC on tumor versus normal tissues, rather than the change in total HLA-I alone. Direct evidence that presentation-restoring therapy causes autoimmunity in pediatric solid tumors is currently lacking; this concern should therefore be framed as a biologically plausible risk requiring prospective testing rather than as an established toxicity. Normal-tissue immunopeptidomics or other target-specific pMHC assays, when technically feasible, are particularly important when a restoration agent is combined with a high-affinity TCR or peptide-centric receptor [11].

From a clinical-risk perspective, two toxicity categories should be distinguished. First, the systemic modulation of cytokine, epigenetic, autophagy, or SUMO pathways can produce class-specific and off-target pharmacologic toxicity independent of tumor recognition. Second, restoration may create on-target/off-tumor immune toxicity if it induces or increases the same therapeutically recognized pMHC on nonmalignant cells, potentially narrowing the safety margin of a high-affinity TCR- or pMHC-directed therapy. The clinical objective should therefore be selective rather than maximal restoration: the lowest effective dose and shortest priming interval, with tumor-local or tumor-targeted delivery where feasible, should be used to increase tumor pMHC while preserving a demonstrable tumor-to-normal presentation margin.

Epigenetic agents illustrate the distinction between early tolerability and long-term therapeutic index. In a pediatric phase I study of entinostat in 21 patients aged 6–20 years with recurrent or refractory solid and CNS tumors, no cycle-1 dose-limiting toxicity was observed at 3 or 4 mg/m^2^ weekly, although grade ≥3 hematologic toxicities occurred and systemic pharmacodynamic activity was measurable in peripheral-blood leukocytes [46]. Thus, pediatric exposure is feasible, but that trial did not test entinostat as an antigen-presentation primer immediately before TCR therapy and cannot establish the safety of increasing tumor and non-tumor presentation simultaneously. For PRC2/EZH2 inhibition, pediatric MATCH treated 20 patients (median age 5 years) with tazemetostat; 30% experienced grade ≥3 adverse events considered at least possibly related to treatment [47]. Long-duration safety is particularly important in children: in 2026, the U.S. Food and Drug Administration announced the voluntary U.S. withdrawal of tazemetostat after an increased rate of hematologic second primary malignancies was identified in a post-approval study [48]. These observations argue against assuming that prolonged epigenetic priming is benign merely because short-course early-phase dosing is tolerable.

Autophagy inhibition likewise lacks pediatric presentation-restoration safety data. Adult phase I experience using hydroxychloroquine as a lysosomal/autophagy inhibitor shows that systemic autophagy inhibition is feasible but can add fatigue, gastrointestinal toxicity, myelosuppression, and other adverse effects; ophthalmologic monitoring was incorporated because of retinal toxicity concerns [49]. These adult data should not be extrapolated to children or to newer pathway-selective agents, and the minimum exposure required for presentation restoration remains undefined. SUMOylation inhibition is even less mature: ML-792 in the osteosarcoma study remains preclinical, while first-in-human adult experience with the SUMO inhibitor subasumstat documented fatigue, nausea and pyrexia, grade ≥3 treatment-related cytopenias and other toxicities, and low-grade cytokine-release syndrome [50]. No pediatric safety window has been established for systemic SUMO-pathway inhibition.

Cytokine-based restoration requires an especially careful separation of tumor-local from systemic effects. Long-term non-oncology experience with IFN-γ in chronic granulomatous disease, including children, reported fever as a common treatment-related event and no discernible adverse effect on growth during prolonged intermittent dosing [51]. This experience is reassuring for the molecule itself but does not validate oncology priming, because cancer dosing, schedule, combination partners, and the intended pharmacodynamic endpoint—broad induction of HLA/APM—differ; the five-patient neuroblastoma pilot [43] is also too small to define a cancer-specific safety window. TNF has a substantially narrower systemic window. A Pediatric Oncology Group phase I study of intravenous recombinant TNF in 27 children with refractory solid tumors reported frequent constitutional toxicity, persistent grade IV hypotension in two patients, transient hepatic dose-limiting toxicity, and one cardiac-arrest death with related complications [52]. Accordingly, the ability of TNF to increase MHC-I/ICAM-1 in sarcoma models [10] should not be interpreted as support for unrestricted systemic TNF administration; local delivery or other tumor-restricted approaches would be more rational if this mechanism is pursued clinically.

Taken together, restoration strategies should advance only if they demonstrate a measurable therapeutic index. A practical development framework should require: (i) confirmation that the intervention increases the intended tumor pMHC rather than merely pan-HLA-I; (ii) assessment of the same pMHC in relevant normal tissues or normal-cell surrogates; (iii) use of the lowest exposure and shortest priming interval that produces the desired tumor pharmacodynamic effect; (iv) preference for local, tumor-targeted, or intermittent delivery when systemic pathway modulation is unnecessary; (v) prespecified monitoring for class-specific acute toxicities and immune-mediated events; and (vi) pediatric long-term follow-up for delayed effects, including secondary malignancies when epigenetic regulators are targeted. For combinations with TCR- or pMHC-directed therapy, the restoration drug and receptor therapy should be treated as a single safety system: an increase in target pMHC on normal tissue could narrow the receptor’s therapeutic window even when each component appears tolerable independently. Safety-window definition is therefore a prerequisite—not an afterthought—for mechanism-matched restoration in children.

## 8. Therapeutic Exploitation of Restored Tumor Visibility

Restoration of antigen presentation is therapeutically meaningful only when the recovered peptide–MHC (pMHC) landscape can be productively recognized. This creates a fundamental distinction between HLA-dependent platforms, which require intact or restored peptide presentation, and MHC-independent platforms, which recognize native cell-surface antigens directly. Therapeutic selection should therefore integrate antigen identity with presentation competence rather than treating them as separate considerations.

### 8.1. TCR-T Therapy: Clinical Proof That pMHC Can Be Therapeutically Actionable

PRAME provides the strongest clinical demonstration of this principle. IMA203 is an autologous TCR-engineered therapy directed against the HLA-A*02:01-restricted PRAME peptide SLLQHLIGL. Mass-spectrometry-based discovery confirmed natural presentation of this epitope on native primary tumors and metastases, whereas it was not quantifiable across 41 normal tissue types. Quantitative analyses further estimated that the transcriptomic eligibility threshold corresponded to approximately 40–50 PRAME peptide–HLA complexes per cell [11]. In a phase 1 study, IMA203 produced objective responses across several advanced PRAME-positive solid tumors, with deeper responses enriched among tumors with higher PRAME expression [11]. These data illustrate that successful TCR therapy requires the convergence of antigen expression, compatible HLA genotype, natural peptide presentation, and sufficient pMHC abundance.

Pediatric proof-of-concept has now emerged in nephroblastoma. In an adolescent with multiply relapsed disseminated disease, high PRAME expression and HLA-A*02:01 positivity were confirmed before the infusion of autologous PRAME-specific TCR-T cells [31]. Treatment was followed by major radiologic, metabolic, and circulating tumor-DNA responses. Although this remains a single-patient experience, it directly demonstrates that an intracellular cancer-testis antigen can be therapeutically exploited through HLA-restricted presentation in pediatric cancer. Representative studies defining the antigen-presentation bottleneck and its therapeutic implications across pediatric solid tumors are summarized in Table 1.

The evidence supporting this framework spans distinct experimental settings and should therefore be interpreted according to the question each study can answer. Berghuis et al. combined patient-tumor immunohistochemistry with in vitro IFN-γ induction experiments in Ewing sarcoma [25], whereas Forloni et al. defined NF-κB-dependent control of MHC-I and ERAP1/2 in mechanistic neuroblastoma cell-line studies [23], and Spel et al. used genome-wide CRISPR screening in neuroblastoma to identify N4BP1 and TNIP1 as regulators of MHC-I display [38]. In osteosarcoma, Tsukahara et al. [26] and Sundara et al. [27] provided patient-tissue observational evidence for context-dependent HLA-I expression, while Reid et al. provided translational and limited clinical evidence that IFN-γ can increase tumor-cell MHC-I and alter T-cell infiltration in high-risk neuroblastoma [43]. These studies contribute complementary rather than equivalent levels of support: human tumor-tissue and clinical observations establish disease relevance, whereas mechanistic and preclinical models define causality, reversibility, and therapeutic plausibility.

### 8.2. Expanding the Actionable pMHC Repertoire

Immunopeptidomics is extending this strategy beyond PRAME. In neuroblastoma, the PHOX2B-derived peptide QYNPIRTTF was identified on HLA-A*24:02 and targeted using peptide-centric CARs that recognize the peptide–HLA complex, rather than native PHOX2B protein. These cells mediated specific neuroblastoma killing and complete tumor regression in murine models [5]. Despite their CAR architecture, such constructs remain HLA dependent because their target is pMHC.

Ewing sarcoma provides a parallel model. Three HLA-A*02:01-restricted LOXHD1 epitopes were identified by immunopeptidomics, enabling the development of TCR353, which mediated Ewing sarcoma killing in vitro and tumor clearance in xenografts. Importantly, cytotoxicity increased after IFN-γ treatment in most models, directly linking enhanced presentation to improved therapeutic activity [7].

Fusion-derived antigens may offer even greater tumor specificity. EWSR1–FLI1-derived peptides have generated T cells capable of recognizing endogenously processed fusion antigen [17]. More recently, vaccination with overlapping fusion-breakpoint peptides induced durable polyfunctional T-cell responses in a patient with metastatic Ewing sarcoma [53]. However, the response was predominantly CD4+, and the contribution of vaccination to prolonged disease stability cannot be separated from prior multimodal therapy, making this supportive rather than definitive evidence of therapeutic MHC-I exploitation.

### 8.3. MAGE-Directed Therapy Reveals Both Opportunity and Escape

MAGE-A4-directed TCR therapy provides broader translational validation. Afamitresgene autoleucel produced objective responses in MAGE-A4-positive solid tumors in phase 1 [54], and activity was subsequently confirmed in the SPEARHEAD-1 phase 2 study of HLA-A*02-positive synovial sarcoma and myxoid round-cell liposarcoma [55].

Resistance in this setting should be separated into three biologically distinct levels: loss or downregulation of the target antigen itself; loss or suppression of the restricting HLA molecule; and dysfunction of downstream antigen-processing and peptide-loading machinery required to generate the MAGE-A4/HLA-A*02 complex. Only the latter two represent presentation defects in the strict sense used in this review, whereas MAGE-A4 loss is target-antigen escape upstream of the presentation bottleneck. This distinction is clinically important because each mechanism implies a different rescue strategy [54,55].

The phase 1 afami-cel data provide direct evidence for target-antigen evolution but do not establish a specific acquired HLA/APM lesion. Two patients who had previously responded received a second afami-cel infusion after progression, and neither responded to retreatment. In one patient, MAGE-A4 expression had fallen markedly between the biopsy obtained before the first infusion and the biopsy obtained before the second infusion (H-score 214 to 37), supporting antigen downregulation as one possible route of escape. In the other patient, however, MAGE-A4 expression remained high before both infusions despite failure of retreatment, demonstrating that persistent antigen expression alone does not establish continued functional pMHC availability or TCR susceptibility [54]. The same phase 1 study also noted that some patients progressed despite persistent circulating afami-cel, further supporting a contribution from tumor-intrinsic resistance rather than loss of the therapeutic cells alone [54].

Importantly, the reported clinical analyses do not identify which HLA/APM component is lost when presentation-associated resistance occurs. The phase 1 study and SPEARHEAD-1 did not establish recurrent acquired loss of the restricting HLA-A*02 allele, HLA-A*02 loss of heterozygosity, B2M loss, or specific loss of TAP1/2, tapasin/TAPBP, ERAP1/2, or immunoproteasome components as a defined resistance mechanism. Although the phase 1 protocol included an exploratory assessment of genes related to antigen processing and presentation, the reported correlative analyses were limited. SPEARHEAD-1 likewise emphasized that the small number of paired pre- and post-infusion biopsies precluded a definitive evaluation of HLA or antigen-presentation loss, and further noted that total HLA-I expression is dynamic and can increase substantially in response to IFN-γ [54,55]. Accordingly, reduced HLA staining after therapy should not be equated automatically with structural HLA loss or a fixed APM defect.

The available clinical observations therefore do not establish whether presentation-associated resistance is reversible. Within the framework proposed here, transcriptional or inflammatory downregulation of an otherwise intact HLA/APM pathway would constitute a soft and potentially reversible defect, whereas acquired loss of the restricting HLA allele or destructive loss of an indispensable component such as B2M would constitute a hard qualitative defect that is unlikely to respond to cytokine or epigenetic restoration. In contrast, loss of MAGE-A4 itself lies upstream of the presentation pathway and would require restoration of target-antigen expression, selection of an alternative antigen, or multi-antigen targeting rather than HLA/APM restoration alone. The afami-cel studies cannot presently distinguish among these possibilities in individual resistant tumors [54,55].

For this reason, resistance analyses in future HLA-restricted TCR-T trials should use paired pretreatment and progression samples to interrogate the pathway in layers: target-antigen expression; integrity and allele-specific expression of the restricting HLA allele; B2M; peptide-processing and loading components such as TAP1/2, tapasin/TAPBP, ERAP1/2 and immunoproteasome subunits; and where technically feasible, the therapeutic pMHC itself. Genomic assessment for structural loss should be combined with expression-level and functional inducibility measurements so that a hard lesion can be distinguished from a soft, potentially restorable state. This approach would convert the currently descriptive observation of ‘HLA/presentation loss’ into a mechanism-defined resistance phenotype that can be matched rationally to restoration, target switching, or MHC-independent bypass.

### 8.4. Bypassing the Bottleneck

When HLA/APM loss is irreversible, conventional CAR-T or antibody-based strategies provide a different route because they recognize native surface proteins independently of peptide processing. GPC2 is a representative oncofetal neuroblastoma target; optimized CAR designs achieved durable tumor control at clinically relevant surface densities of approximately 1000–6000 molecules per cell [13]. Ewing sarcoma surface proteomics has similarly identified ENPP1 as a membrane-enriched candidate with relatively restricted pediatric normal-tissue expression, although clinical validation is still lacking [56].

The therapeutic consequence of the antigen-presentation bottleneck is therefore clear: tumors with intact but suppressed presentation machinery may be rendered susceptible to TCR- or pMHC-directed therapies, whereas tumors with irreversible HLA/APM loss require MHC-independent recognition of native surface antigens. Antigen selection and presentation competence must therefore be evaluated together.

## 9. Precision Biomarkers and a Future Therapeutic Framework

The antigen-presentation bottleneck has a central implication for precision immunotherapy: antigen expression and presentation competence should be evaluated as a single therapeutic phenotype. An antigen-positive tumor cannot be assumed to be susceptible to HLA-restricted therapy because defects in HLA integrity, antigen processing, peptide loading, or surface pMHC abundance may render an otherwise attractive antigen functionally inaccessible. Patient selection should therefore move beyond expression-based biomarkers toward an integrated assessment of the complete antigen-presentation axis.

### 9.1. Integrated Assessment of Antigen Availability and Presentation Competence

For HLA-dependent therapies, evaluation should begin with high-resolution HLA typing and confirmation that the therapeutically relevant allele remains intact. Pan-pediatric profiling demonstrates that HLA loss of heterozygosity and locus-specific transcriptional suppression can coexist with apparently preserved global HLA-I expression, making pan-HLA immunohistochemistry alone insufficient to establish presentation competence [1]. Assessment should therefore incorporate allele-specific HLA expression together with key antigen-processing components such as B2M, TAP1/2, ERAP proteins, and regulatory nodes including NLRC5.

For longitudinal resistance monitoring, baseline and progression biopsies should therefore be evaluated with the same layered logic. In addition to confirming continued target-antigen expression, testing should distinguish constitutional HLA eligibility from tumor-acquired change by assessing the restricting HLA allele for copy-number/LOH events and allele-specific expression, together with B2M and core processing/loading components. Where sufficient material is available, paired genomic and expression analyses should be complemented by direct or targeted pMHC measurement, and in experimental systems, inducibility testing after IFN-γ or another mechanism-matched perturbation. This is particularly important for interpreting afami-cel resistance, because the clinical studies documented MAGE-A4 downregulation in one resistant setting but did not resolve a recurrent HLA-A*02, B2M, or downstream APM lesion [54,55].

Antigen abundance represents a complementary, but distinct, biomarker layer. PRAME immunohistochemistry, for example, identifies substantial protein expression across neuroblastoma specimens [57], yet expression alone cannot establish processing into a therapeutically relevant HLA ligand. This distinction is reinforced by medulloblastoma immunopeptidomics, where precursor RNA and protein abundance correlated incompletely with naturally presented HLA peptides and substantial interpatient variation was observed [6]. Transcriptomic and proteomic assays should therefore function principally as target-enrichment tools, whereas direct immunopeptidomic confirmation provides stronger evidence that an intracellular antigen is genuinely accessible to HLA-dependent therapy.

### 9.2. Quantitative pMHC as a Functional Biomarker

Where the relevant HLA allele and core APM components remain available, presentation should also be regarded as a quantitative rather than purely binary variable. In the PRAME-004 program, mass spectrometry confirmed natural presentation of the target peptide on native tumors, while quantitative analyses linked the transcriptomic eligibility threshold to approximately 40–50 peptide–HLA complexes per cell [11]. This illustrates a more informative biomarker paradigm in which pMHC density is interpreted relative to the recognition threshold of the therapeutic receptor.

Such an approach is particularly important because successful T-cell recognition depends not only on whether a peptide is presented, but also on how much is displayed and how sensitively the therapeutic receptor detects it. Biomarker development should therefore integrate antigen abundance, HLA genotype, HLA allelic integrity, APM competence, natural peptide presentation, and quantitative pMHC density rather than relying on any single variable.

### 9.3. A Presentation-Guided Therapeutic Taxonomy

These variables can be integrated using two independent axes. The first is phenotypic: quantitative presentation failure, in which the relevant pMHC is present but below a functional recognition threshold, versus qualitative presentation failure, in which a required HLA/APM element is absent or functionally unavailable and the relevant pMHC cannot be generated or displayed. The second is mechanistic: soft defects, in which the pathway remains structurally intact and potentially restorable, versus hard defects, in which structural loss or other fixed disruption limits rescue. The axes are complementary rather than synonymous.

This distinction changes therapy selection. A quantitative-soft phenotype may be approached by increasing presentation or preventing MHC-I/pMHC loss before TCR- or pMHC-directed therapy. A qualitative-soft phenotype—for example, profound but reversible silencing of a required HLA/APM component—requires reconstitution of the missing function before HLA-dependent therapy can be expected to work. In contrast, a hard qualitative defect such as tumor-specific loss of the restricting HLA allele is unlikely to be rescued pharmacologically and favors MHC-independent recognition of native surface antigens. Hard structural abnormalities may also impose a quantitative ceiling when presentation capacity is reduced rather than completely abolished; in that setting, functional pMHC measurement is more informative than total HLA staining alone.

The afami-cel experience provides a useful clinical illustration of this taxonomy. MAGE-A4 loss represents target-level escape rather than an antigen-presentation defect; retained MAGE-A4 with regulatory HLA/APM suppression would represent a soft presentation bottleneck; acquired structural loss of the restricting HLA-A*02 allele or an indispensable component such as B2M would represent a hard qualitative presentation defect; and retained antigen with apparently preserved HLA but absent or insufficient target pMHC would direct attention to downstream processing, loading, trafficking, or pMHC-density limitations. Because the available phase 1 and SPEARHEAD-1 biopsies did not resolve these pathway states at progression, the resistant tumors cannot be assigned retrospectively to a specific quadrant of the framework without additional paired molecular and functional data [54,55].

Accordingly, classification should proceed in sequence: (1) confirm the target antigen; (2) determine whether the relevant pMHC is absent or merely quantitatively insufficient; (3) localize the limiting HLA/APM component; and (4) determine whether that lesion is reversible. This framework shifts precision therapy away from asking whether a tumor merely “expresses” an antigen and toward identifying the specific presentation phenotype that must be restored—or bypassed—for that antigen to become therapeutically visible.

### 9.4. Spatial and Longitudinal Profiling

A single pretreatment biopsy may provide an incomplete representation of presentation competence. Pediatric tumors can display substantial intratumoral heterogeneity, and recurrence may be accompanied by biological states that differ materially from those present at diagnosis. Large-scale pediatric brain-tumor proteogenomics has demonstrated substantial molecular and proteogenomic differences between initial and recurrent/progressive specimens and shown that protein-level consequences of genomic alterations are not always evident from transcriptomic data alone [58]. In this context, spatial sampling is not merely a descriptive issue: if HLA-I-positive and HLA-I-low/negative compartments coexist, a biopsy can be biologically valid for the sampled region yet unrepresentative of the presentation phenotype across the whole tumor [27,28].

Pediatric immune-profiling studies remain limited, and the application and integration of advanced multiplex techniques have been sparse, constraining multidimensional characterization of childhood tumors [59]. Future biomarker programs should therefore integrate genomic, transcriptomic, proteomic, spatial, and immunopeptidomic measurements longitudinally, particularly before and after presentation-restoring or T-cell-directed therapy.

Accordingly, spatial profiling should be incorporated into biomarker validation rather than reserved only for future descriptive studies. In tumors with heterogeneous HLA/APM staining, multiregion sampling, spatially resolved immunohistochemistry or digital pathology, and—where technically feasible—allele-specific HLA/APM assays or region-matched immunopeptidomics can help determine whether the relevant presentation phenotype is homogeneous or compartment-restricted. Longitudinally, paired diagnostic/relapse and pre-/post-treatment samples are needed to distinguish stable structural loss from acquired or reversible suppression and to detect treatment-driven evolution of presentation competence.

For biomarker-guided HLA-restricted therapy, the practical consequence is that eligibility should be interpreted with explicit sampling uncertainty. A single biopsy may be sufficient when presentation markers are spatially uniform, but discordant regional findings should prompt broader sampling or orthogonal validation before concluding that a target pMHC is consistently available—or consistently absent—across the disease burden.

The clinically relevant endpoint is ultimately not whether a tumor once expressed a candidate antigen, but whether that antigen is being presented at sufficient density, in the relevant tumor compartment, at the time therapy is delivered. Thus, presentation competence should be defined in both space and time, not inferred from a single historical specimen.

## 10. Limitations and Evidence Gaps

### 10.1. Biological and Evidence-Base Gaps

The evidence supporting an antigen-presentation bottleneck is uneven across pediatric solid tumors. Mechanistic studies are concentrated in neuroblastoma, Ewing sarcoma, osteosarcoma, and medulloblastoma, whereas rhabdomyosarcoma, nephroblastoma, and rarer developmental tumors remain substantially less characterized. This imbalance is important because pediatric solid tumors arise from diverse cellular and developmental contexts and cannot be assumed to share equivalent mechanisms of immune invisibility [60]. Moreover, substantial mechanistic evidence for reversible MHC-I suppression and restoration derives from adult tumor models, and its prevalence and therapeutic relevance in pediatric malignancies require direct validation [61,62]. The targeted disease-level review summarized in Section 5.6 identified direct presentation-related evidence in Wilms tumor [30], retinoblastoma [32], hepatoblastoma [33], AT/RT [34], and DMG [35], while the germ-cell-tumor evidence [36] derives from a mixed-age cohort rather than a pediatric-only series. These datasets range from small historical tissue series to cell-line or xenograft studies and are not equivalent in evidentiary strength. The disease-specific sections are therefore intended as evidence-weighted mechanistic exemplars rather than a claim of uniform or systematic coverage across all pediatric solid tumors. Dedicated pediatric antigen-presentation studies remain particularly limited for ependymoma and several rarer entities. Accordingly, PRC2-mediated silencing [39], SUMO-dependent APM repression [18], pancreatic autophagic MHC-I degradation [42], and chronic interferon-associated resistance [45] were used in this review as cross-age or cross-tumor mechanistic frameworks unless pediatric-specific validation was cited separately; none were taken as evidence of prevalence or clinical effect in childhood tumors.

### 10.2. Methodological Limitations

A major methodological challenge is that antigen expression is not equivalent to antigen presentation. Transcriptomic, proteomic, serological, and immunohistochemical approaches can nominate candidate targets but cannot establish natural pMHC display; medulloblastoma immunopeptidomics demonstrates incomplete correspondence between precursor abundance and the HLA ligandome [6]. Earlier neuroblastoma antigen-discovery approaches similarly identified immunogenic candidates without directly demonstrating therapeutically relevant pMHC presentation [63]. Limited tissue availability, small cohorts, single-marker assays, and intratumoral heterogeneity further constrain pediatric immune profiling [59].

Clinical translation of immunopeptidomics is constrained by both sample input and analytical sensitivity. Pediatric core or needle biopsies may provide only a few milligrams of tissue and can have low tumor cellularity, while HLA immunoprecipitation, peptide elution, cleanup, and sample transfer each introduce material loss. Rare therapeutic pMHC species may therefore remain below the detection threshold even when biologically present. Conventional data-dependent acquisition (DDA) adds a further limitation because stochastic precursor selection favors more abundant ions and reduces sensitivity and run-to-run reproducibility in low-input material. Consequently, failure to detect a candidate pMHC in a small biopsy should be interpreted as a sensitivity-limited negative result rather than definitive evidence of biological absence. Data-independent acquisition (DIA) partially addresses this problem by systematically sampling predefined m/z windows; multi-HLA spectral-library DIA has produced substantially greater peptide identification than DDA while preserving specificity, making it particularly attractive when clinical material is scarce [64].

Several technical developments may make clinical immunopeptidomics more feasible and scalable. Automated microfluidic immunoaffinity purification minimizes transfers and sample loss and has recovered more than 4000 HLA-I peptides from 0.2 million cells and more than 5000 from only 5 mg of tumor tissue [65]. Sensitive single-shot PASEF-based acquisition can eliminate offline fractionation and has identified more than 800 HLA-I peptides from approximately 1 million cells while supporting the high-throughput analysis of tumor-derived material [66]. For routine clinical deployment, however, sensitivity alone is insufficient. Pre-analytical variables—including tissue mass, tumor cellularity, ischemia/storage conditions and HLA genotype—must be recorded alongside the capture antibody, peptide-recovery controls, LC-MS performance, search space, false-discovery-rate criteria, and the analytical limit of detection. Reference materials, system-suitability testing, and validation of accuracy, precision, specificity, limit of detection, and robustness are needed to improve inter-run and inter-laboratory comparability [67]. When a predefined therapeutic pMHC is being used for patient selection, confirmation with synthetic isotope-labeled peptide standards or an orthogonal target-specific assay can further strengthen confidence. Thus, a clinically scalable workflow is likely to combine low-input automated preparation with reproducible DIA or sensitive single-shot acquisition, standardized quality control, and harmonized reporting rather than relying solely on discovery-scale DDA.

### 10.3. Translational and Clinical Gaps

Prospective pediatric evidence linking pharmacologic restoration of MHC-I to improved TCR- or pMHC-directed clinical efficacy remains limited. Standardized longitudinal assessment of HLA integrity, APM competence, natural peptide presentation, and quantitative pMHC density will therefore be required before presentation-guided treatment can be implemented routinely. In parallel, MHC-independent target discovery is expanding: age-stratified pediatric profiling identified CLDN6 as a developmentally restricted native surface antigen with strong expression in selected pediatric solid tumors, particularly germ cell tumors and desmoplastic small round cell tumors, while expression in normal tissues largely diminished after early postnatal life [68]. Because this review is narrative rather than systematic, its synthesis is also inherently susceptible to literature-selection bias. Routine implementation will also require analytically validated low-input immunopeptidomic workflows with harmonized pre-analytical handling, acquisition, quality control, and reporting, as outlined in Section 10.2.

Future resistance studies should also be designed explicitly to distinguish target loss from presentation escape. Paired pretreatment, on-treatment, and progression tissue should be collected whenever feasible, and progression after HLA-restricted TCR therapy should trigger the staged assessment of target-antigen expression, restricting-HLA integrity and allele-specific expression, B2M/APM status, and functional target-pMHC display rather than the attribution of resistance generically to ‘HLA loss’. Where the pathway is structurally intact but suppressed, ex vivo inducibility can help identify a potentially restorable soft defect; where structural loss is demonstrated, restoration strategies should give way to target switching or MHC-independent approaches. The limited paired-biopsy analyses in the afami-cel studies illustrate why this distinction should be built prospectively into future clinical protocols [54,55].

The major translational challenge is consequently not simply to identify additional antigens, but to demonstrate prospectively that restoration of functional pMHC display produces durable antigen-specific therapeutic benefit in children.

Safety is an equally important translational gap. Prospective pediatric studies should define the minimum exposure required to produce a tumor-specific presentation response, incorporate on-treatment pharmacodynamic measurements in tumor and accessible normal tissues, and capture both acute class-specific toxicity and late effects. Existing early-phase and regulatory evidence documents class-specific acute and long-term safety concerns across the restoration approaches discussed above [46,47,48,49,50,51,52]. For combinations with TCR- or pMHC-directed therapy, immune-mediated and cytokine-associated toxicities should additionally be monitored prospectively because the combination-specific safety profile remains insufficiently defined.

## 11. Conclusions

Pediatric solid tumors should not be regarded simply as antigen-poor malignancies. Many retain developmentally restricted, cancer-testis, fusion-derived, or lineage-associated antigens, yet these targets may remain immunologically inaccessible because of defective processing and MHC-I presentation. The central therapeutic barrier may therefore lie not only in antigen scarcity, but in a failure to convert intracellular antigen abundance into sufficient surface peptide–MHC display.

This distinction creates a practical therapeutic framework. Tumors with genetically intact but suppressed antigen-presentation machinery may be rendered susceptible to TCR- or pMHC-directed therapy through mechanism-matched restoration, whereas irreversible disruption of HLA-I or essential APM components favors deliberate transition to MHC-independent surface targeting. Future therapeutic development should therefore assess antigen availability and presentation competence together, rather than treating antigen discovery as an independent endpoint. The central question for pediatric T-cell immunotherapy is therefore not only whether a target antigen reaches sufficient pMHC-I density for effective recognition, but whether any presentation failure is quantitative or qualitative and whether the causative lesion is soft/potentially reversible or hard/structural. These distinctions determine whether tumor visibility can be restored for HLA-dependent therapy or must instead be bypassed. Accordingly, a biologically restorable bottleneck is therapeutically useful only if restoration can be achieved within a pediatric-appropriate safety window that preferentially increases tumor visibility without creating clinically relevant normal-tissue pMHC exposure.

## Figures and Tables

**Figure 1:**
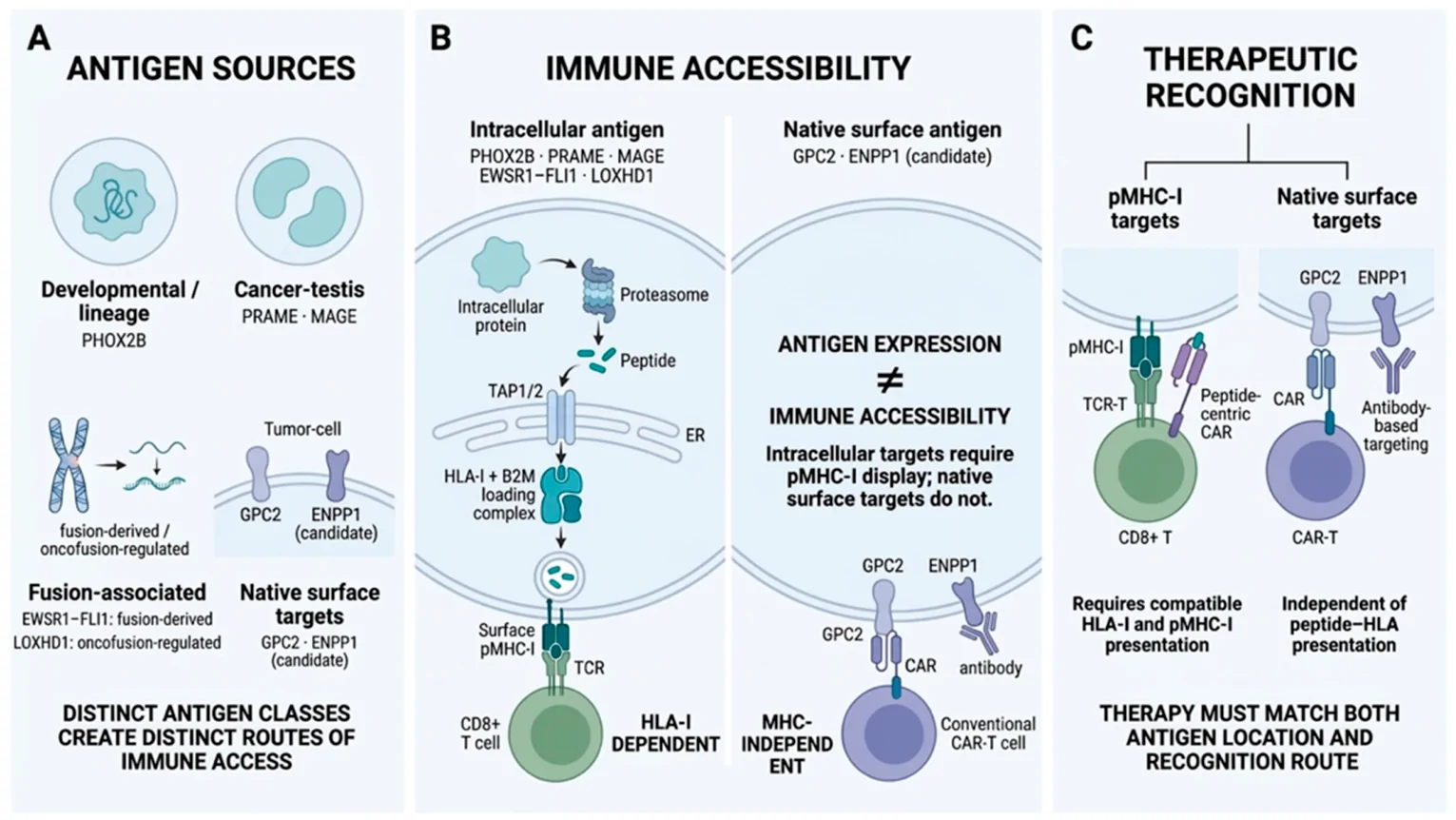
Antigen sources, immune accessibility, and therapeutic recognition in pediatric solid tumors. (**A**) Representative antigen classes include developmental/lineage, cancer-testis, fusion-associated, and native surface targets. (**B**) Intracellular antigens require proteasomal processing, TAPmediated transport, HLA-I/B2M loading, and surface pMHC-I display for TCR recognition, whereas native surface antigens are directly accessible independently of peptide–HLA presentation. (**C**) These distinct recognition routes determine therapeutic accessibility: TCR-T and peptide-centric CAR approaches target pMHC-I, whereas conventional CAR-T and antibody-based strategies recognize native surface antigens directly.

**Table 1:** Representative studies defining the antigen-presentation bottleneck and its therapeutic relevance in pediatric solid tumors.

Study	Tumor/Setting	Evidence Level/Setting	Study Design and Material	Key Findings	Relevance to the Antigen-Presentation Bottleneck
Lim et al., 2024 [1]	Recurrent/refractory pediatric and AYA solid tumors	Patient-cohort observational/translational	Integrated immunogenomic analysis of 576 patients across 11 tumor cohorts using paired WES and RNA-Seq; HLA-I expression additionally assessed in 194 tumors and 44 PDXs	Somatic HLA/APM mutations were uncommon overall, whereas HLA LOH and transcriptional suppression were more prominent. HLA-I expression was heterogeneous, and loss was frequently associated with reduced HLA-B and TAP expression.	Establishes the pan-pediatric landscape of genetic and transcriptional presentation failure and supports distinction between structural and potentially reversible defects.
Velz et al., 2025 [6]	Medulloblastoma	Patient-tissue translational/immunopeptidomic	Integrative multi-omics and HLA immunopeptidomics of 28 native tumors spanning WNT, SHH, Group 3, and Group 4, with functional testing of prioritized peptides	Identified extensive naturally presented HLA-I and HLA-II repertoires, but most tumor-associated peptides were incompletely shared across molecular subgroups. Precursor expression did not reliably predict presentation.	Provides direct evidence that antigen abundance and natural HLA presentation are non-equivalent and demonstrates the value of immunopeptidomics.
Yarmarkovich et al., 2023 [5]	Neuroblastoma	Translational preclinical; patient tissue + in vivo	Genomic and immunopeptidomic profiling of 8 xenografts and 8 primary high-risk neuroblastomas, followed by peptide-centric CAR engineering and preclinical validation	Identified the PHOX2B-derived QYNPIRTTF–HLA-A*24:02 complex as a naturally presented lineage-restricted target. PHOX2B peptide-centric CARs mediated specific tumor killing and complete regression in murine models.	Demonstrates that an intracellular developmental oncoprotein becomes therapeutically accessible through pMHC presentation.
Forloni et al., 2010 [23]	Neuroblastoma	Preclinical mechanistic; in vitro	Human neuroblastoma cell-line studies examining NF-κB regulation of MHC-I and the peptide-trimming aminopeptidases ERAP1 and ERAP2	NF-κB p65 directly regulated MHC-I expression, and manipulation of NF-κB altered both MHC-I and ERAP1/2, demonstrating coordinated regulation of antigen processing and presentation.	Establishes upstream transcriptional control as a mechanism through which multiple elements of the MHC-I/APM pathway can be coordinately suppressed rather than independently lost.
Lorenzi et al., 2012 [22]	Aggressive neuroblastoma	Preclinical mechanistic + patient-tissue support	Mechanistic cell-line studies with IRF1 and NF-κB p65 manipulation, tumor-tissue immunohistochemistry, and antigen-specific CTL assays	Combined IRF1/NF-κB reconstitution restored multiple MHC-I/APM components and rendered neuroblastoma cells susceptible to MAGE-A3-specific CTL killing.	Defines coordinated transcriptional suppression as a prototypical reversible presentation defect and demonstrates restoration of functional antigen recognition.
Spel et al., 2018 [38]	Neuroblastoma	Preclinical mechanistic; in vitro functional genomics	Genome-wide CRISPR-based screening with functional validation in multiple neuroblastoma cell lines	Identified N4BP1 and TNIP1 as suppressors of NF-κB-driven MHC-I expression; their depletion increased surface MHC-I and improved antigen-specific CD8+ T-cell recognition.	Identifies actionable upstream regulators of a reversible transcriptional presentation defect and provides functional evidence that restoring MHC-I can improve T-cell recognition.
Cornel et al., 2022 [8]	High-risk neuroblastoma	Preclinical translational; in vitro and organoid	High-throughput drug-repurposing screen followed by validation in neuroblastoma cell lines and patient-derived organoids, with PRAME-specific T-cell and NK-cell coculture	HDAC inhibition, particularly entinostat, increased MHC-I, TAP1/2 and immunoproteasome components, enhanced immune-cell cytotoxicity, and induced a more immunogenic lineage state.	Provides pediatric evidence that epigenetic and lineage-associated MHC-I suppression is pharmacologically reversible.
De Mitri et al., 2025 [9]	Neuroblastoma	Preclinical; in vitro and in vivo	Human and murine neuroblastoma models, 3D spheroids, immune coculture, and in vivo experiments using genetic and pharmacologic autophagy inhibition	Autophagy inhibition increased surface MHC-I, promoted activated CD8+ T-cell infiltration, reduced tumor growth, and enhanced T-cell and GD2-CAR-T antitumor activity.	Supports post-translational regulation of tumor visibility and identifies autophagy as a therapeutically reversible mechanism.
Reid et al., 2009 [43]	High-risk neuroblastoma	Translational/pediatric clinical pilot	Preclinical neuroblastoma studies combined with systemic IFN-γ exposure in a pilot cohort of five patients with high-risk neuroblastoma	IFN-γ increased tumor-cell MHC-I, enhanced susceptibility to tumor-specific T-cell recognition, and promoted T-cell infiltration. In the five-patient pilot, two patients showed tumor MHC-I upregulation accompanied by increased CD3+ T-cell infiltration, whereas the remaining tumors showed limited responses.	Provides direct pediatric translational evidence that low MHC-I can remain inducible in vivo while also demonstrating interpatient heterogeneity in restoration capacity.
Berghuis et al., 2009 [25]	Ewing sarcoma	Patient-tissue observational + preclinical in vitro	HLA-I assessment in 67 Ewing sarcoma specimens together with IFN-γ stimulation of Ewing sarcoma cell lines and analysis of APM components	Complete or partial HLA-I absence was observed in 79% of tumors, with lung metastases consistently lacking detectable HLA-I. IFN-γ induced TAP1/2, tapasin, immunoproteasome components, B2M, and HLA-I in cell lines.	Demonstrates that clinically observed HLA-I loss in Ewing sarcoma can coexist with an inducible, structurally retained presentation pathway, supporting a potentially reversible defect.
Blanchard et al., 2025 [7]	Ewing sarcoma	Translational preclinical; immunopeptidomic + in vivo	Computational epitope prediction, HLA-A*02:01 immunopeptidomics, TCR isolation, cell-line assays, and xenograft validation; LOXHD1 expression additionally assessed in patient transcriptomic data	Three naturally presented LOXHD1-derived HLA-A*02:01 epitopes were identified. TCR353-engineered CD8+ T cells killed Ewing sarcoma cells and achieved tumor clearance in xenograft models; presentation increased after IFN-γ in selected models.	Demonstrates that an oncofusion-regulated, non-mutated antigen can generate actionable pMHC targets and links enhanced presentation to therapeutic activity.
Tsukahara et al., 2006 [26]	Osteosarcoma	Patient-tissue observational/prognostic	Immunohistochemical assessment of HLA-I expression in primary and metastatic osteosarcoma specimens with clinical outcome analysis	HLA-I loss or downregulation was reported in 52% of primary tumors and 88% of metastatic lesions; higher HLA-I expression was associated with better overall and event-free survival.	Provides patient-level evidence that HLA-I loss is common in osteosarcoma and may increase with progression, supporting clinical relevance of presentation competence.
Sundara et al., 2017 [27]	Osteosarcoma	Patient-tissue observational/spatial	Analysis of HLA-I expression and tumor-infiltrating lymphocyte localization in primary and metastatic osteosarcoma tissue	HLA-I expression was frequently retained, and tumor-infiltrating T cells spatially colocalized with HLA-I-positive tumor regions.	Demonstrates spatial coupling between HLA-I expression and immune infiltration and supports intratumoral heterogeneity as an important determinant of biopsy interpretation and patient selection.
Yu et al., 2026 [24]	Osteosarcoma	Preclinical mechanistic; in vitro and in vivo	Integrated molecular analyses with mechanistic osteosarcoma models and pharmacologic targeting of the SUMO pathway	UBA2 promoted SUMO2-dependent SESN2 modification, enhanced autophagic flux, and drove NBR1-mediated MHC-I degradation; UBA2 inhibition restored immune visibility and improved immunotherapeutic responsiveness.	Defines a SUMOylation–autophagy–MHC-I degradation axis linking post-translational regulation to immune escape in osteosarcoma.
Mair et al., 2026 [31]	Multiply relapsed pediatric nephroblastoma	Clinical; single-patient proof-of-concept	Named-patient use of autologous PRAME-specific TCR-T in one 17-year-old patient with PRAME-high, HLA-A*02:01-positive disseminated disease	Treatment produced substantial radiologic and metabolic regression together with a marked molecular response, including clearance of detectable circulating tumor DNA.	Provides direct pediatric clinical proof-of-concept that an intracellular cancer-testis antigen can be therapeutically exploited through HLA-restricted presentation.
Wermke et al., 2025 [11]	PRAME-positive, HLA-A*02-positive advanced solid tumors	Clinical; phase I	First-in-human multicenter open-label phase 1 IMA203 TCR-T trial; 27 patients in dose escalation and 13 in dose extension; 40 patients received IMA203	Unconfirmed/confirmed ORR was 52.5% (21/40), with confirmed ORR of 28.9% (11/38). Deeper responses were enriched with higher PRAME expression, and quantitative analyses linked eligibility to low but measurable target pMHC abundance.	Provides clinical validation that successful TCR therapy depends on convergence of antigen expression, compatible HLA, natural presentation, and sufficient pMHC density.

Note: Evidence-level/setting designations are descriptive and indicate the principal experimental context of each study (patient-tissue observational, in vitro mechanistic, in vivo preclinical, translational, or clinical); they are not intended as a formal quality-of-evidence grading system. Studies combining more than one evidence type are labeled accordingly. Abbreviations: APM, antigen-processing machinery; AYA, adolescent and young adult; CTL, cytotoxic T lymphocyte; HDAC, histone deacetylase; HLA, human leukocyte antigen; LOH, loss of heterozygosity; MHC-I, major histocompatibility complex class I; NK, natural killer; ORR, objective response rate; PDX, patient-derived xenograft; pMHC, peptide–MHC complex; TAP, transporter associated with antigen processing; TCR-T, T-cell receptor-engineered T-cell therapy; WES, whole-exome sequencing.

## Data Availability

No new data were created or analyzed in this study. Data sharing is not applicable to this article.
